# Overexpression of Terpenoid Biosynthesis Genes Modifies Root Growth and Nodulation in Soybean (*Glycine max*)

**DOI:** 10.3390/cells11172622

**Published:** 2022-08-23

**Authors:** Mohammed Ali, Long Miao, Fathia A. Soudy, Doaa Bahaa Eldin Darwish, Salma Saleh Alrdahe, Dikhnah Alshehri, Vagner A. Benedito, Million Tadege, Xiaobo Wang, Jian Zhao

**Affiliations:** 1 Egyptian Deserts Gene Bank, North Sinai Research Station, Desert Research Center, Department of Genetic Resources, Cairo 11753, Egypt; 2 College of Agronomy, Anhui Agricultural University, Hefei 230036, China; 3 Department of Genetics and Genetic Engineering, Faculty of Agriculture, Benha University, Moshtohor, Toukh 13736, Egypt; 4 Botany Department, Faculty of Science, Mansoura University, Mansoura 35511, Egypt; 5 Department of Biology, Faculty of Science, University of Tabuk, Tabuk 71491, Saudi Arabia; 6 Plant and Soil Sciences Division, Davis College of Agriculture, Natural Resources and Design, West Virginia University, Morgantown, WV 26506, USA; 7 Department of Plant and Soil Sciences, Institute for Agricultural Biosciences, Oklahoma State University, Ardmore, OK 73401, USA; 8 State Key Laboratory of Tea Plant Biology and Utilization, College of Tea and Food Science and Technology, Anhui Agricultural University, Hefei 230036, China

**Keywords:** *Glycine max*, *Salvia guaranitica*, root growth and nodulation, strigolactone, terpenoid synthesis gene

## Abstract

Root nodule formation in many leguminous plants is known to be affected by endogen ous and exogenous factors that affect formation, development, and longevity of nodules in roots. Therefore, it is important to understand the role of the genes which are involved in the regulation of the nodulation signaling pathway. This study aimed to investigate the effect of terpenoids and terpene biosynthesis genes on root nodule formation in *Glycine max*. The study aimed to clarify not only the impact of over-expressing five terpene synthesis genes isolated from *G. max* and *Salvia guaranitica* on soybean nodulation signaling pathway, but also on the strigolactones pathway. The obtained results revealed that the over expression of *GmFDPS*, *GmGGPPS*, *SgGPS*, *SgFPPS*, and *SgLINS* genes enhanced the root nodule numbers, fresh weight of nodules, root, and root length. Moreover, the terpene content in the transgenic *G. max* hairy roots was estimated. The results explored that the monoterpenes, sesquiterpenes and diterpenes were significantly increased in transgenic soybean hairy roots in comparison with the control. Our results indicate the potential effects of terpenoids and terpene synthesis genes on soybean root growth and nodulation. The study provides novel insights for understanding the epistatic relationship between terpenoids, root development, and nodulation in soybean.

## 1. Introduction

Soybean (*Glycine max*) is considered one of the oldest polyploidy (pa leopolyploid) plants and one of the most domesticated food crops in the world; it is expected to contribute to sustainable agriculture through its ability for symbiotic nitrogen fixation [1]. The symbiotic interaction between soybean roots and *B. japonicum* bacteria, leads to the formation of unique structures known as root nodules. Hosted inside the root nodule, rhizobia can transform the molecular nitrogen gas (N_2_) from atmosphere into ammonia (NH_3_), which will be readily available to the plant, and for this exchange of benefits deal, rhizobia are amended with plant carbohydrates [1,2]. Various factors regulate root nodule formation such as certain plant hormones, some metabolic enzymes, and definite transcription factors from the approach of the nodulation signal all the way to nodule initiation, development, and maturation [3,4]. Furthermore, several genes related to secondary metabolism (e.g., Phenylpropanoids, terpenoid and isoflavonoids biosyntheses) were identified by microarray analysis from *Lotus japonicu* nodule with higher frequency in nodule parenchyma (NP) and nodule vascular bundle (NC), compared with un-nodulated root [5]. Previously, we found that, the knockdown of *MtHMGR1* gene form Medicago plant (*Medicago truncatula*) led to a decrease in nodules formation, which is considered a key gene in the Mevalonate (MVA) pathway that can interact with the DMI2 gene for induced symbiotic interaction and nodule development. Moreover, the use of RNA interference (RNAi) tool for silencing both genes *GmMAX1a* and *GmMAX4a* led to a dramatic decrease in nodule numbers in soybean plants. Recently, we found that the overexpression of *SoTPS6, SoNEOD, SoLINS, SoSABS, SoGPS,* and *SoCINS* genes from *Salvia officinalis* in soybean hairy roots, produces a drastic increase in root growth and nodulation [6].

Terpenoids components with various structures and sizes are considered one from the largest ecophysiologically active secondary metabolites [6,7,8,9,10]. Legumes are one of the most vital foodstuffs worldwide: leguminous species (e.g., soybean, snow pea, lentil, lupine mung bean, hairy vetch, alfalfa, medicago, white clover, and red clover,) produce various secondary metabolites, such as isoflavones and terpenoids compounds, which play role(s) as auxin transport regulators, plant defense, plant growth, acting as signals to regulate the symbiotic interaction of legume plants and rhizobia [11,12,13,14,15,16,17,18,19]. Various studies have shown that the terpenoids could induce the expression of Nod factor and nod signaling genes such as, *GmNRF1α*, *GmNRF5α*, *GmNSP1α*, *GmNSP2α*, *GmDMI2α* and *GmDMI3β* in soybean [6]. Moreover, strigolactones (SLs) are a class of hormones found predominantly in many plants such as, Arabidopsis, Pea, Rice, Petunia, and soybean [6,19,20,21,22,23]. Strigolactones (SLs) have different physiological roles that are correlated to root growth and development, branching of shoot, and mycorrhiza and formation of root nodules in legume [23,24,25]. Earlier studies have illustrated that strigolactones genes that were detected in root of soybean and alfalfa seedlings are involved in enhanced nodulation by inducing the expression of Nod genes in rhizobial bacteria [4,23,26,27].

Over the years, *Agrobacterium rhizogenes*-mediated transformation of soybean (*G. max*) hairy roots has become a powerful agent to investigate the responsibility of genes that are concerned in root biological roles such as plant–microbe communication, nutrient uptake, and hormone transport [6,28]. We have successfully used this system to clarify the role of various genes such as: *GmMAX1a*, *GmMAX4a* and *GmIMaTs*, that are involved in soybean nodulation [27,29]. Here, we characterized five genes from *G. max* and *S. guaranitica* that are involved in terpenoid and terpene biosynthesis, and determined theirbiological role in the interaction with rhizobia and promotion of nodulation in *G. max*. The inclusion methodologies that were employed to reach this goal are the following: (i) overexpression of *GmFDPS*, *GmGGPPS*, *SgGPS*, *SgFPPS*, and *SgLINS* genes in the domesticated soybean hairy roots; (ii) investigating nodulation and root phenotypes at 10 and 20 days after *B. japonicum* inoculation; (iii) profiling terpenoid in transgenic *G. max* hairy roots by GC-mass; and (iv) monitoring the transcription of genes implicated in nodulation signaling and strigolactones biosynthesis by qPCR. In the context that, an important question has been raised: what are the key roles of terpenoid genes in root development and nodulation? This question was difficult to answer before conducting the present work because there was a lack of information at the genetic level regarding the terpenoid biosynthetic pathway and the roles of these genes in root development and nodulation. Interestingly, we may be able to answer the question through our findings, which support the significance of the previous terpenoid genes in rhizobial infection by elucidating the associations between the overexpression of terpenoid, nodulation-signaling, and strigolactone-biosynthesizing genes.

## 2. Materials and Methods

### 2.1. In-Silico Differntial Gene Expression and Phylogenetic Analysis

Phylogenetic tree was created via MEGA6 using the Neighbor-Joining method with 1000 bootstraps. *G. max* and *S. guaranitica* terpenoid biosynthetic pathway genes and the deduced amino acid sequences were searched using RNA-Seq Data Analysis and Phytozome database (phytozome.jgi.doe.gov) accessed on 12 April 2021 and identified soybean proteins with high sequence similarity (≥90% normalized identity) to annotated terpenoid biosynthesis genes of *G. max* and *S. guaranitica* through BlastP. Moreover, to investigate the putative accumulated transcript of *GmFDPS*, *GmGGPPS*, *SgGPS*, *SgFPPS*, and *SgLINS* across nine different tissues, we used public RNA-Seq meta-analyses from multifarious studies were presented from the Atlas of soybean (http://bar.utoronto.ca/eplant_soybean/, accessed on 25 March 2021). Additionally, *GmFDPS*, *GmGGPPS*, *SgGPS*, *SgFPPS*, and *SgLINS* predicted subcellular localization was inferred from its Arabiposis homologous genes as retrieved from the Arabidopsis Information Resource (https://phytozome.jgi.doe.gov/pz/portal.html#!info?Alias=Org_Athaliana, accessed on 25 March 2021). Ultimately, the image that showed the subcellular localization was built using Cell Electronic Fluorescent Pictograph Browsers (Cell eFP: http://bar.utoronto.ca/cell_efp/cgi-bin/cell_efp.cgi, accessed on 25 March 2021).

### 2.2. Cloning of Full-Length Terpenoid Synthase cDNAs

The *GmFDPS*, *GmGGPPS*, *SgGPS*, *SgFPPS*, and *SgLINS* full-length cDNAs were amplified with short and long gene-specific primers designed based on our transcriptome sequencing of *S. guaranitica* leaves as well as the soybean database (https://phytozome.jgi.doe.gov/pz/portal.html, accessed on 21 March 2021) (Table S1). Polymerase Chain Reaction (PCR) reaction was performed using short primers, KOD-Plus DNA polymerase enzyme (Toyobo, Japan) and leaf cDNA as a template with the following program (94 °C: 3 min, 98 °C: 10 s, 57 or 60 °C: 30 s, 68 °C: 1.5 min, 68 °C: 10 min, and 35 cycles), for the first PCR. For the second PCR we used the first PCR products as at templates, long primers and the same previous compounds and program. Afterwards, the PCR products were purified, cloned into the pDONR221 Gateway entry vector, and then subcloned into the pB2GW7 Gateway destination vector as described earlier [6,8,9,10]. Following this, the destination vector was used to introduce our previous genes into the *G. max* hairy roots via *Agrobacterium rhizogenes*-K599 by electroporation. Sanger sequencing was used to verify the success of the cloning steps. The soybean hairy root transformation and rhizobial inoculation were performed as described earlier [6]. Briefly, soybean seeds were sterilized and germinated in sterilized vermiculite under controlled conditions. Hereafter, the bacterial suspension of the recombinant *A. rhizogenes* was prepared accordingly and was injected into the hypocotyl proximal of vigorous soybean seedlings with flattened cotyledons. Then, the transformed seedlings were grown under suitable and controlled conditions. On the tenth day from root transformation, rhizobial inoculation by *B. japonicum* USDA-110 was properly performed in ten to twelve replicates. Finally, transformed plants with properly established hairy roots and nodules were harvested for photographing or further differential gene expression analyses by qRT-PCR.

### 2.3. Differential Gene Expression by qRT-PCR

According to the manufacturer’s methods and instructions, total RNA was extracted from different biological replicates using Trizol Reagent (Invitrogen, Carlsbad, CA, USA). The extracted RNA was treated with DnaseI (Takara, Beijing, China), and its integrity was checked using 1.2% agarose-formaldehyde gel and with ethidium bromide staining. However, its purity and concentration were analyzed by NanoDrop™ 2000/2000c Spectrophotometers (Wilmington, MA, USA). For either cloning or qRT-PCR, cDNA synthesis was performed with a reverse transcription kit (M-MLV, Beijing, China) using 10 µg of RNA [6,8,9,10]. To elucidate the differential gene expression of terpenoid biosynthesis, Strigolactone biosynthesis and early nodulation signaling genes (Tables S2 and S3) across different treatments, iQTM5 Multicolor Real-Time PCR Detection System (Bio-Rad, Agitech, New Cairo Cairo, Egypt) with SYBR Green fluorescence (and ROX as a passive reference dye; Newbio Industry, Beijing, China) was used according to [6,8,9,10]. The primers were designed via the IDTdna tool (https://eu.idtdna.com/scitools/Applications/RealTimePCR/, accessed on 12 June 2021), listed in (Tables S2 and S3). To calculate the cycle threshold (CT) of the target genes, GmActin was used as a reference gene to normalize the gene expression levels. Finally, the delta delta Ct method was used to calculate relative gene expression levels [30].

### 2.4. Quantitative GC-MS of Terpenoids

Fresh in vitro hairy roots from either *GmFDPS-OE*, *GmGGPPS-OE*, *SgGPS-OE*, *SgFPPS-OE*, and *SgLINS-OE* or GUS lines were promptly frozen using liquid nitrogen. Then, powder samples were soaked in 10 mL of n-hexane and incubated with shaking at 37 °C and 200 rpm for 72 h as described earlier [6]. Afterwards, the supernatant was concentrated to 1.5 mL, and transferred to fresh crimp 1.5-mL vial amber glass. The vials were then placed on the GC-MS auto-sampler. Following that, the quantification of terpenoids was done via GCMS-QP2010 Ultra (Shimadzu, Tokyo, Japan) with HP-5 fused silica capillary column (30 m × 0.25 mm ID, 0.25 µm film thicknesses), Helium gas at flow rate 1.0 mL/min and 1-µL aliquot injection volume. We used n-Hexadecane (CAS Number: 544-76-3; https://www.sigmaaldrich.com/EG/en/product/mm/820633, accessed on 25 March 2021) as an internal standard. Finally, the type and relative % concentration for each component was determined by comparison of their mass spectra with the mass spectra data were that stored in the various Libraries, as previously described by [6,8,9,10].

### 2.5. Statistical Analyses

Soybean hairy root measurements were analyzed by the Student’s *t*-test to estimate the effects of gene overexpression and time on the root length (cm), fresh root weight (gram), fresh nodule weight (gram) and nodule numbers, and compared to the control roots (*GUS*-overexpressing hairy roots). Each column represents the mean ± SD of the parameter, and statistical significance was based on the Student’s *t*-test (* *p* < 0.05; ** *p* < 0.01; n.s., not significant) with GUS-overexpressing hairy roots as control.

Gene accession numbers: *Salvia guaranitica* geranyl diphosphate synthase (*SgGPS*, KX893917); farnesyl pyrophosphate synthetase (*SgFPPS*, KX893918); (3S)-linalool synthase (*SgLINS*, KX893965). *Glycine max* farnesyl diphosphate synthase (*GmFDPS*, XP_003534984.1); geranylgeranyl diphosphate synthase (*GmGGPP*, XP_003537515.2).

## 3. Results

### 3.1. Identification of Terpenoid Biosynthesis Genes from Soybean and Sage Plants

With a focus on the putative biosynthetic genes in the soybean genome, we managed a BLASTP search against the soybean genome using functionally characterized of *S. guaranitica* terpenoid biosynthesis proteins as queries. This approach identified several proteins closely related to *SgGPS*, *SgFPPS*, and *SgLINS* genes. These sequences were submitted to phylogenetic analysis (Figure S1). The putative expression patterns of terpenoid biosynthesis genes of soybean were uncovered by transcript analysis across nine tissues using the Phytozome database (phytozome.jgi.doe.gov/). Interestingly, we observed the highest expression levels of these genes in root hairs, roots, and nodules (Figure S2). In plants, there are two pathways responsible for terpene biosynthesis: the plastidial (MEP, methylerythritol 4-phosphate: MD:M00096) pathway; and the cytosolic (MVP, mevalonate: MD:M00095) pathway that produces different terpenoids (e.g., monoterpenes, sesquiterpenes, diterpenes, triterpenes, carotenoids, and sterols) [31,32,33]. Moreover, interconnection exists between SLs, nodulation signaling molecules and terpene since all of these compounds are derived from terpenoids/isoprenoids. For that reason, we closelyinvestigated the prospective subcellular localization for these genes, relying upon Arabidopsis protein localization to identify the probable synthesis sites using the Cell eFP browsers (http://bar.utoronto.ca/cell_efp/cgi-bin/cell_efp.cgi). From this analysis, the previous genes are localized mainly to the cytosol, mitochondrion, nucleus, and plastid (Figure S3).

### 3.2. Overexpression of Terpenoid Genes Changed Soybean Root Growth

**Figure 1:**
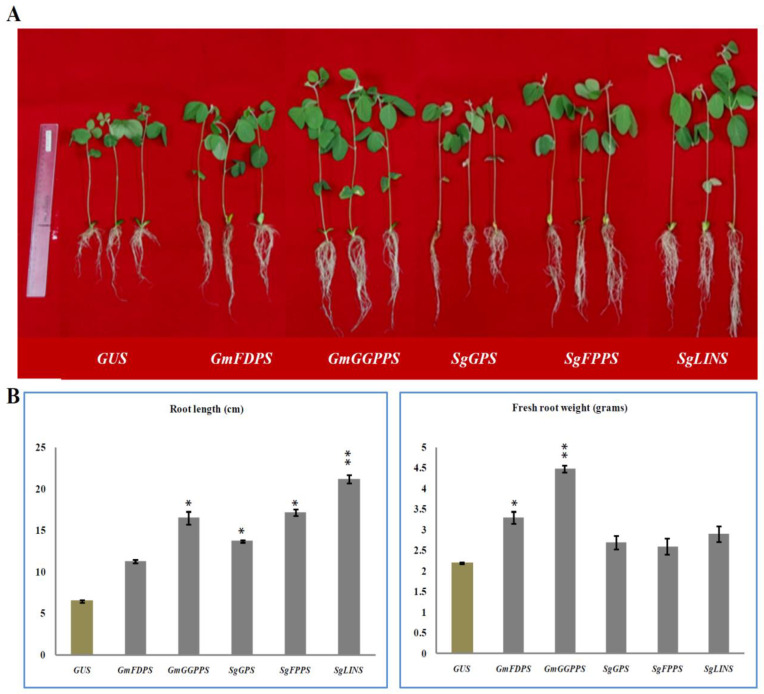
Effects of terpenoid gene overexpression on root and shoot growth. (**A**) Representative photos of chimerical *G. max* plants with transgenic hairy roots overexpressing terpene synthesis genes. Chimerical soybean plants were generated by transformation with K599 harboring terpene genes including (left to right), GUS (control), *GmFDPS, GmGGPPS, SgGPSS, SgFPPS* and *SgLINS*. (**B**) In vivo hairy roots’ fresh weight (gram) and root length (cm). Root phenotypes were examined for at least 10 independent lines (*n* = 10). Each column represents the mean ± SD of the parameter and statistical significance was based on the Student’s *t*-test (* *p* < 0.05; ** *p* < 0.01) with GUS-overexpressing hairy roots as control.

To evaluate the effect of *GmFDPS*, *GmGGPPS*, *SgGPS*, *SgFPPS*, and *SgLINS* genes on soybean root phenotypes after25days without inoculation (non-nodulating), these genes were cloned from *G. max* and *S. guaranitica* and over-expressed in soybean hairy root as a transgenic expression system. The stable constitutive overexpression of those genes in hairy roots were carried out by the infection of *G. max* green seedling cotyledons using *A. rhizogenes* carrying pB2GW7-*GmFDPS*, pB2GW7-*GmGGPPS*, pB2GW7-*SgGPS*, pB2GW7-*SgFPPS* and pB2GW7-*SgLINS* under the control of 35S promoter. Transgenic hairy roots were successfully generated, which have higher root length and fresh weight than the GUS control (Figure 1A,B). These results are in line with Ali et al. [6] and Samudin and Kuswantoro [34] who found the overexpression of terpenoid genes in soybean roots led to higher fresh root weight and length compared to control in cases without inoculation by *B. japonicum*, which confirmed the decisive role of terpenoids genes in soybean root development [6]. In previous reports, various TPSs family genes were highly coordinated in root and cell-specific processes, such as: marneral; β-amyrin and thalianol synthesis as a triterpene; rhizathalene synthase (*AtTPS08*) as diterpene; (Z)-γ-bisabolene synthases as a sesquiterpene; and 1,8-cineole synthase as a monoterpene [35,36,37,38,39,40,41,42]. These previous genes were co-expressed primarily in the root epidermis cells, the stele of the root elongation, differentiation/maturation zones, epidermis and cortex of older roots and other different root tissues for producing a “superhairy” different root phenotype [35,36,37,38,39,40,41,42]. These previous reports and results demonstrated the role of TPSs family genes in root growth and development [6].

### 3.3. Overexpression of Terpene Synthase Genes Changed the Terpene Profiles in Transgenic Soybean Hairy Roots

**Figure 2:**
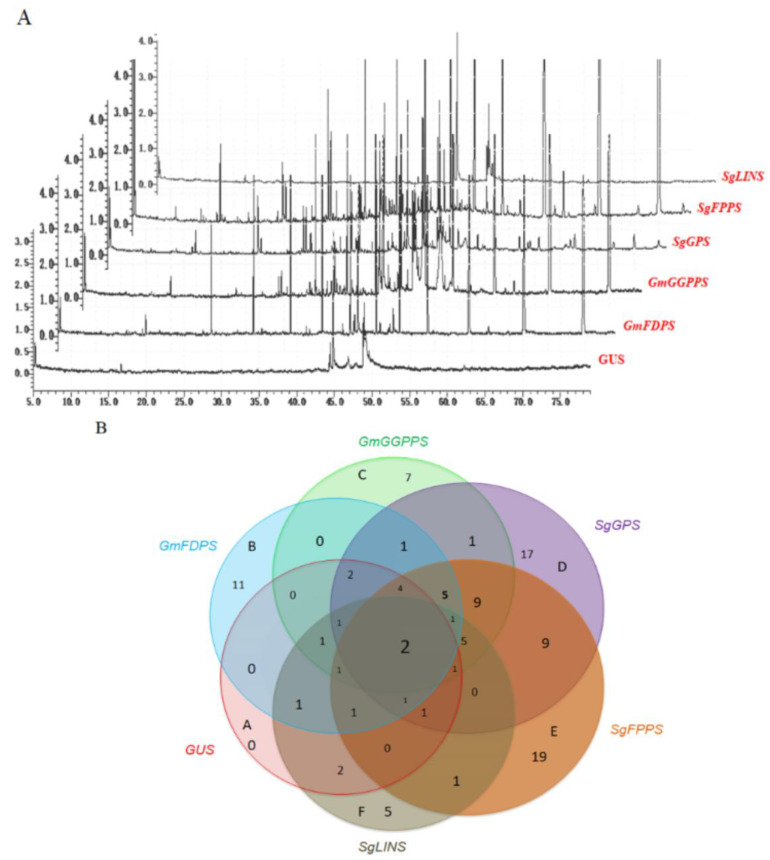
Typical GC-MS mass spectragraphs for terpenoids from six hexane extracts from the different overexpression of terpenoid biosynthesis genes. (**A**) GC-MS Peak of the essential oil; (**B**) Six-way Venn diagram to show the number of unique and common compounds in the essential oil extracts from GUS (A), *GmFDPS* (B), *GmGGPPS* (C), *SgGPS* (D), *SgFPPS* (E) and *SgLINS* (F) of *G. max* and *S. guaranitica*.

To explore the consequence of overexpression of terpenoid biosynthesis genes (e.g., GUS as a control, *GmFDPS*, *GmGGPPS*, *SgGPS*, *SgFPPS*, and *SgLINS*) in transgenic *G. max* hairy roots. GC-Mass was performed to analyze the qualitative and quantitative changes in the terpene profiles in transgenic *G. max* hairy roots. The analysis confirmed that various terpene profiles were significantly increased in transgenic hairy roots overexpressing terpene synthetic genes compared with GUS as reported in Table 1 and Figure 2A.

In roots overexpressing *GmFDPS*, sesquiterpenes represented the main compounds (11.87%), followed by monoterpenes (10.5%) and one diterpene compound (9.62%). In roots overexpressing *GmGGPPS* and *SgFPPS*, diterpenes were the major group accountingfor 12.09 and 13.0%, respectively, followed by monoterpenes (4.29 and 3.47%), and sesquiterpenes (0.25 and 1.72%). Meanwhile, roots overexpressing *SgGPS* produced monoterpene (6.12%), followed by sesquiterpenes (4.83%) and one diterpene compound (1.11%). On the other hand, monoterpenes (1.4%) were observed as the major compound category in roots overexpressing *SgLINS*.

Moreover, the six hexane extracts from the different overexpression of terpenoid biosynthesis genes (e.g., GUS as a control, *GmFDPS, GmGGPPS, SgGPS, SgFPPS*, and *SgLINS*), have unique, common, and major phytochemical compounds (Table 1 and Figure 2A). For example, the extracts of *GmFDPS* essential oils (B) had 11 unique compounds, two common compounds shared with extracts from *SgGPS* essential oils, one common compound shared with extracts from *SgFPPS*, one common compound shared with extracts from *GmGGPPS* and *SgLINS*, one common compound shared with extracts from *GmGGPPS* and *SgFPPS*, one common compound shared with extracts from *SgGPS* and *SgLINS*, one common compound shared with extracts from *GmGGPPS*, *SgGPS, SgFPPS* and *SgLINS* essential oils (Table 1 and Figure 2B). Furthermore, the *GmGGPPS* essential oils (C) contained seven unique compounds, one common compound shared with extracts from *SgGPS*, five common compounds shared with extracts from *SgFPPS*, one common compound shared with extracts from *SgLINS*, nine common compounds shared with extracts from *SgGPS* and *SgFPPS* essential oils. Moreover, the extracts from *SgGPS* essential oils (D) had 17 unique compounds, nine common compounds shared with extracts from *SgFPPS* essential oils.

Moreover, the extracts from *SgFPPS* essential oils (E) and the *SgLINS* essential oils (F) had 19 and five unique compounds, respectively (Figure 2B). On the other hand, we found two common compounds named ((8) Annulene and Isomenthol) shared with all six extracts.

### 3.4. Overexpression of Terpenoid Biosynthesis Genes after Soybean Hairy Root Nodulation

**Figure 3:**
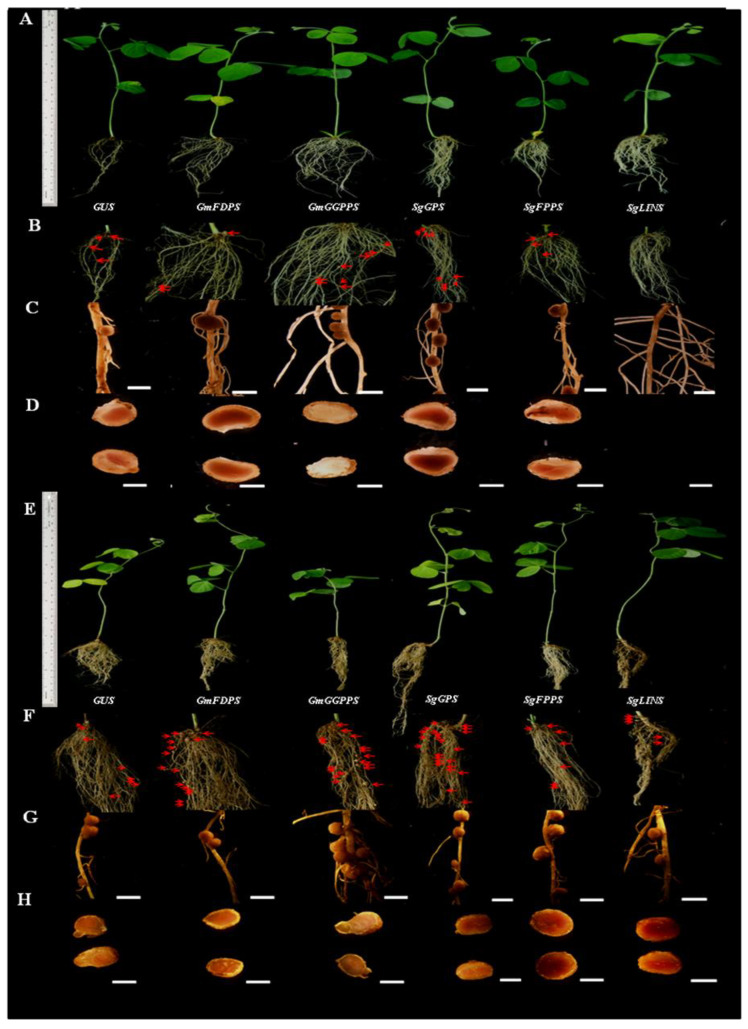
Effect of terpenoid gene overexpression on soybean root nodulation. Roots and nodules were examined at 10th and 20th day after rhizobia inoculated with *B. japonicum* strain USDA110. Composite plants were generated by transformation with the K599 vector harboring overexpression cassettes for *GUS* (control), *GmFDPS, GmGGPPS, SgGPS, SgFPPS* and *SgLINS*. Roots were inoculated with rhizobia. (**A**,**E**) Root and shoot phenotypes of 10- and 20-d-old *G. max* plants. (**B**,**F**) Locations where nodules formed on hairy roots overexpressing 10- and 20-days after rhizobial inoculation. (**C**,**G**) Nodules developed on secondary roots. (**D**,**H**) Cross-sections of *G. max* nodules. Photographs in (**C**,**D**,**G**,**H**) were taken with a DP-73 microscope camera set (Olympus, Tokyo, Japan). Scale bars in (**C**,**D**,**G**,**H**) = 500 μm.

**Figure 4:**
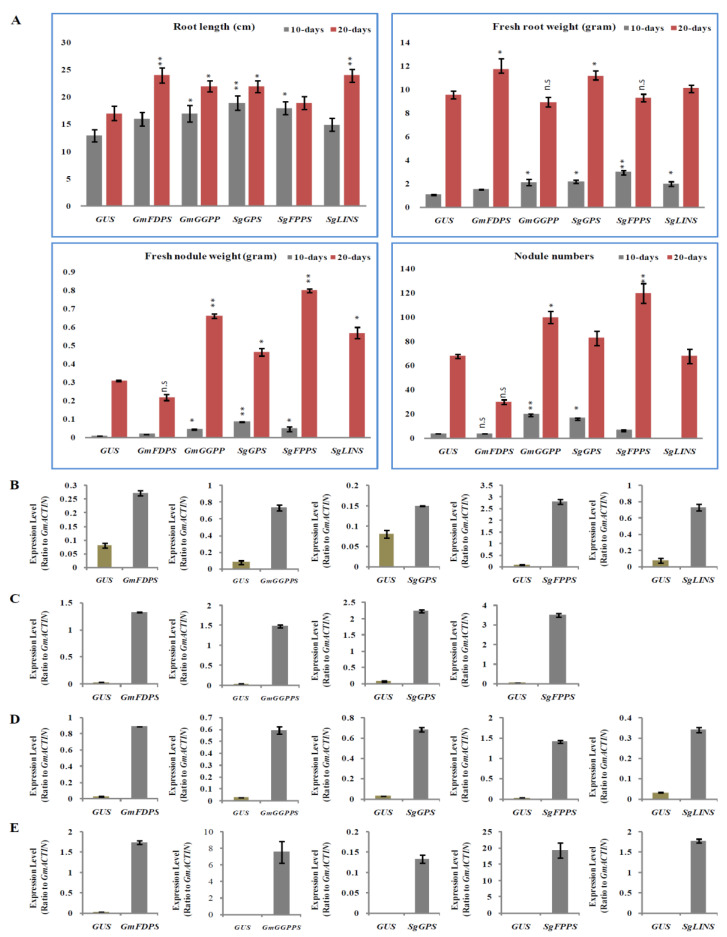
Effects of terpenoid synthesis gene overexpression on root growth and nodule development at 10 and 20 days of rhizobia inoculation. (**A**) In vivo root length (cm), fresh root weight (gram), fresh nodule weight (gram) and nodule numbers, were examined (*n* = 10–12). Blue and red columns represent the effect of gene overexpression after 10 and 20 days after rhizobial inoculation. Data are presented as means ± SD and statistical significance is based on Student’s *t*-test (* *p* < 0.05; ** *p* < 0.01; (n.s.), not significant) with GUS-overexpressing hairy roots as the control; (**B**) Quantitative RT-PCR for in vivo hairy roots after 10days from *B. japonicum* (USDA110) infection. The error bars indicate the SD of three qRT-PCR biological replicates; (**C**) qRT-PCR for nodules after 10days from infection; (**D**) qRT-PCR for in vivo hairy roots after 20days from infection; (**E**) qRT-PCR for nodules after 20days from infection.

Five genes from *G. max* and *S. guaranitica* were cloned and overexpressed in soybean roots, then soybean roots were inoculated with *B. japonicum* (USDA110), to explore the effects of these genes on soybean root phenotypes and nodulation after 10 and 20 days Figure 3A–H. The following root and nodule characteristics were investigated: root length (cm), fresh root weight (gram), fresh nodule weight (gram) and nodule number after 10 and 20 days, as indicated in Figure 4A. *GmFDPS, GmGGPPS, SgGPS, SgFPPS*, and *SgLINS* expression were validated in roots and nodules after 10 and 20days by qRT-PCR, with substantial overexpression compared with GUS-containing plants (Figure 4B–E). Our results reveal that overexpression of *GmFDPS, GmGGPPS, SgGPS, SgFPPS*, and *SgLINS* led to a significant increase in root length and fresh root weight after 10 days. Moreover, the overexpression of *GmFDPS*, *GmGGPPS*, *SgGPS*, *SgFPPS*, and *SgLINS* led to a significant increase in root length after 20days, while only the overexpression of *GmFDPS* and *SgGPS* led to a significant increase in fresh root weight after 10days (Figure 4A). Furthermore, when compared to GUS lines, overexpression of the *GmGGPPS*, *SgGPS*, and *SgFPPS* resulted in higher nodule counts and dramatically increased nodules fresh weight for a given amount of root after 10 and 20 days (Figure 4A). On the contrary, the overexpression of *SoLINS* after 10 days from inoculation led to formation of a few numbers from ultra-fine unmature nodules with meager fresh weight compared to the GUS. Our results are in agreement with [6,43] who found the nodule grow and formation at soybean and japans cultivars peanut need for a longer period, which means that there is a suitable and standard diameter of the 1st-order lateral roots for nodule formation that related with each growth stage. Besides this, we investigated the effect of *GmFDPS*, *GmGGPPS*, *SgGPS*, *SgFPPS*, and *SgLINS* overexpression after 20 days from infection and validated these previous genes overexpression in transgenic hairy roots and nodules using qRT-PCR (Figure 4D–E). Our findings suggest that the *GmFDPS*, *GmGGPPS*, *SgGPS*, *SgFPPS*, and *SgLINS* genes may play a short and long-term function in the soybean nodulation signaling pathway after 10 and 20 days from *B. japonicum* infection.

### 3.5. Relative Expression Analysis of Nodulation and Strigolactone Biosynthesis Genes in Transgenic Soybean Hairy Roots at 10 DAI by B. japonicum

**Figure 5:**
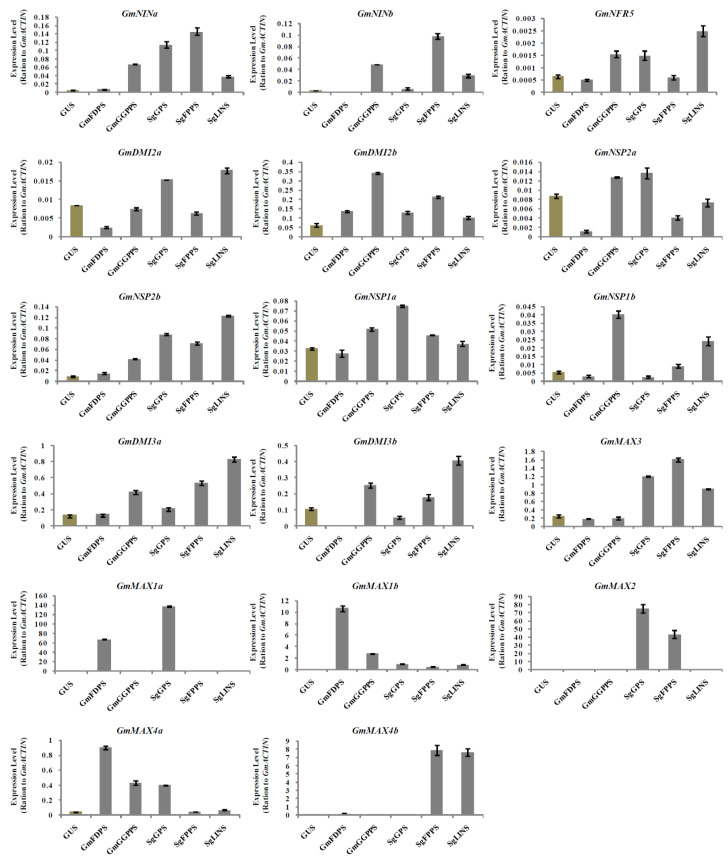
Expression profiles of SL biosynthesis and nodulation genes in soybean transgenic hairy roots after 10 days of rhizobia inoculation. Relative gene expression was analyzed using quantitative real-time PCR compared to *GUS* as a control. The housekeeping *GmB-ACTIN* gene was used as an internal reference gene for expression normalization. The error bars indicate the SD of three qRT-PCR biological replicates.

The creation of root nodules is mediated by successful communication between the legume root and the rhizobia, which is established by transmitting chemical signals from both sides to recognize one other and initiate the infection thread [44]. Therefore, the successful production of these chemical signals is crucial for nodulation. These signals are biosynthesized by specific genes; for example, the early nodulation signaling genes such as *GmNINa*, *GmNINb*, *GmNRF5*, *GmDMI2a*, *GmDMI2b*, *GmNSP2a*, *GmNSP2b*, *GmNSP1a*, *GmNSP1b*, *GmDMI3a* and *GmDMI3b* and SL biosynthetic genes such as *GmMAX3*, *GmMAX1a*, *GmMAX1b*, *GmMAX2*, *GmMAX4a*, and *GmMAX4b*. These previous genes are well-known to control the biosynthesis of these chemical signals. At 10-DAI, the chosen seventeen early nodulation signaling, and SL biosynthetic genes were expressed differently in the transgenic hairy roots (Figure 5). For example, the expression levels of *GmNSP2a*, *GmNSP1a*, *GmMAX1a*, and *GmMAX2*, were highest in hairy roots overexpressing *SgGPS*. *GmMAX1b* and *GmMAX4a* transcription levels were markedly increased in hairy roots overexpressing *GmFDPS*, while the highest expression levels for *GmNINa*, *GmNINb*, *GmMAX3*, and *GmMAX4b* were observed in hairy roots overexpressing *SgFPPS*. Moreover, *GmNRF5*, *GmDMI2a*, *GmNSP2b*, *GmDMI3a*, and *GmDMI3b* were at the highest expression levels in hairy roots overexpressing *SgLINS* (Figure 5). Additionally, the impact of *GmFDPS*, *GmGGPPS*, *SgGPS*, *SgFPPS*, and *SgLINS* genes overexpression on the expression of nodulation signaling genes and SLs biosynthesis in nodules at 10 DAI by *B. japonicum*, were investigated to determine whether it plays a role during rhizobial infection, early phases of nodule formation, and nodule growth.

**Figure 6:**
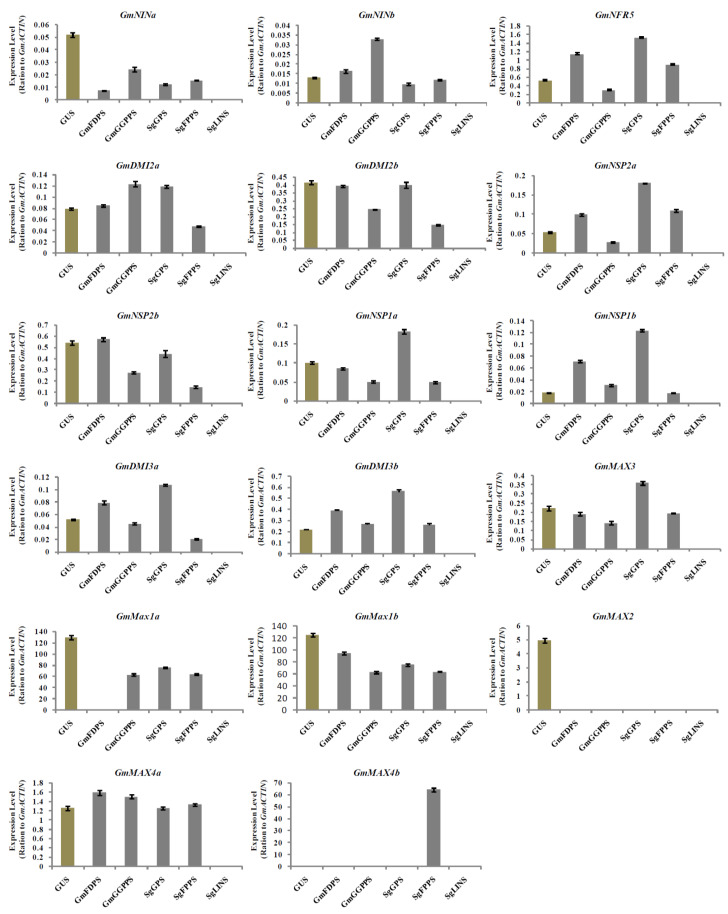
Expression profiles of SL biosynthesis and nodulation genes in soybean nodules after 10 days of rhizobia inoculation.Gene expression was analyzed using quantitative real-time PCR as compared to *GUS* as a control. The housekeeping *GmB-ACTIN* gene was used as an internal reference gene for expression normalization. The error bars indicate the SD of three qRT-PCR biological replicates.

The results show that the previously mentioned list of selected genes was differently expressed in nodules at 10 DAI (Figure 6). Intriguingly, the expression of *GmNSP2b* and *GmMAX4a* were highest in nodules overexpressing *GmFDPS*. On the other hand, *GmNINb* and *GmDMI2a* transcription levels were markedly increased in nodules by overexpressing *GmGGPPS*. Moreover, the highest expression levels for *GmNRF5*, *GmNSP2a*, *GmNSP1a*, *GmNSP1b*, *GmDMI3a*, *GmDMI3b*, and *GmMAX3* were observed in nodules overexpressing *SgGPS*. Additionally, *GmMAX4b* expression was highest in nodules overexpressing *SgFPPS* (Figure 6). The effect of the *GmFDPS*, *GmGGPPS*, *SgGPS*, *SgFPPS*, and *SgLINS* transgene on these genes’ expressions in the transgenic hairy roots and nodules after 10 days of *B. Japonicum* infection, concludes the involvement of our genes in nodule formation. This finding suggests that tepene synthese genes perform important functions during nodulation signaling and early nodule development by controlling the transcription of the main genes responsible for nodulation.

### 3.6. Relative Expression Analysis of Nodulation and Strigolactone Biosynthesis Genes in Transgenic Soybean Hairy Roots at 20 DAI by B. japonicum

**Figure 7:**
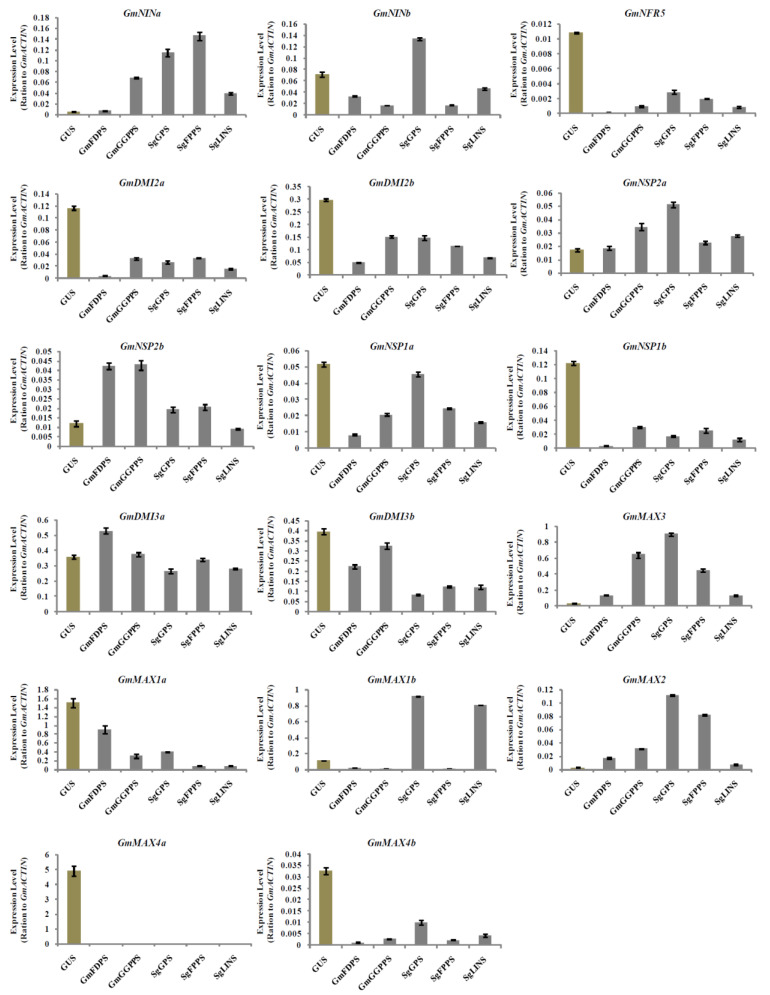
Expression profiles of SL biosynthesis and nodulation genes in soybean transgenic hairy roots after 20 days of rhizobia inoculation. Gene expression was analyzed using quantitative real-time PCR compared to *GUS* as a control. The housekeeping *GmB-ACTIN* gene was used as an internal reference gene for expression normalization. The error bars indicate the SD of three qRT-PCR biological replicates.

**Figure 8:**
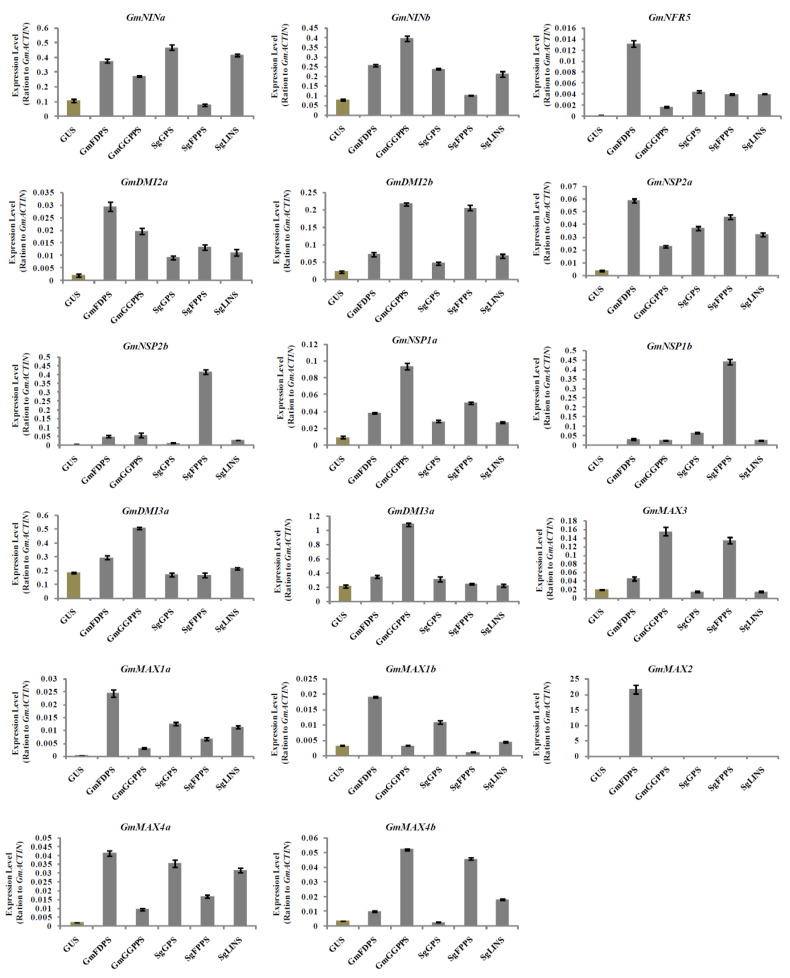
Expression profiles of SL biosynthesis and nodulation genes in soybean nodules after 20 days of rhizobia inoculation. Gene expression was analyzed using quantitative real-time PCR compared to *GUS* as a control. The housekeeping *GmB-ACTIN* gene was used as an internal reference gene for expression normalization. The error bars indicate the SD of three qRT-PCR biological replicates.

Investigating the impact of *GmFDPS*, *GmGGPPS*, *SgGPS*, *SgFPPS*, and *SgLINS* genes overexpression on the expression of nodulation signaling and SLs biosynthesis genes in soybean hairy roots and nodules at 20 DAI may better understand the long-term influence on root and nodule development. Therefore, the expression analysis of the selected genes involved in nodulation signaling and SLs biosynthesis were analyzed by qRT-PCR.The results showed that the nodulation signaling, and SLs biosynthesis genes were upregulated in hairy roots and nodules at 20 DAI (Figure 7 and Figure 8). For example, the expression of *GmDMI3a* was highest in hairy roots overexpressing *GmFDPS*, while the highest level of *GmNSP2b* transcription was observed in hairy roots overexpressing *GmGGPPS*. Moreover, the expression levels of *GmNINb*, *GmNSP2a*, *GmMAX3*, *GmMAX1b* and *GmMAX2* were highest in hairy roots overexpressing *SgGPS.* Furthermore, *GmNINa* expression was highest in hairy roots overexpressing *SgFPPS* (Figure 7). In addition, expression of *GmNRF5*, *GmDMI2a*, *GmNSP2a*, *GmMAX1a*, *GmMAX1b*, *GmMAX2*, and *GmMAX4a* were the highest in nodules overexpressing *GmFDPS.* The highest expression levels for *GmMAX3*, *GmMAX4b*, *GmNINb*, *GmNSP1a*, *GmDMI2b*, *GmDMI3a*, and *GmDMI3b* were observed in nodules overexpressing *GmGGPPS*. Besides, *GmNINa* expression was highest in nodules overexpressing *SgGPS*, while *GmNSP2b* transcription was highest in nodules overexpressing *SgFPPS* (Figure 8). Consequently, these data indicate that the terpenoid biosynthesis genes expression orchestrates nodulation signaling and SL biosynthesis genes in hairy roots and nodules at 20 DAI.

## 4. Discussion

### 4.1. Characterization, Putative Expression Patterns, and Subcellular Localization of Terpenoid Genes from Soybean and Anise-Scented Sage Plants

Cultivated soybean is the one of the oldest sources of vegetable oils worldwide and one of the world’s most significant food crops. Soybean terpenoid includes many mono-, sesqui-, di-, Sester-, tri-,sesquar- and tetraterpenes components, incorporate linalool, Cis-verbenol, α-pinene, Limonene dioxide, trans-ocimene, α-humulene, a-Terpineol, (E, E)-α-farnesene, Farnesan, Dihydrophytol, Phytol, b,b-Carotene, Squalene [1,6]. Only a few recent studies have described the role and function of terpenoid genes in soybean root development and nodulation [6,45,46,47,48]. In this study, we used a BLAST algorithm to find the terpenoid biosynthesis genes in the cultivated soybean genome. The putative terpenoid biosynthesis-encoding genes from *G. max* and anise-scented sage (*S. guaranitica*, Lamiaceae) were used as queries.

Phylogenetic analysis showed that the close homolog to *GmFDPS*, *GmGGPPS*, *SgGPS*, *SgFPPS*, and *SgLINS* from *G. max* and *S. guaranitica* were Glyma.02G059000.1, Glyma.19G144800.1, Glyma.05G100400.4, and Glyma.15G121400.1, respectively (Figure S1). To identify *GmFDPS*, *GmGGPPS*, *SgGPS*, *SgFPPS*, and *SgLINS* biological functions, their expression patterns in nine different tissues based on their increased resemblance to genes from *G. max* were identified and predicted. The results show that their constitutively expressed for all of these genes are mostly expressed in (root_hairs, root and nodules) (Figure S2). Forexample, the expression levels of Glyma.02G059000.1, Glyma.05G100400.4, and Glyma.15G121400.1 genes were highest in all the nine tissues, especially in nodules, root, and root_hairs. Therefore, their expression patterns are similar to other orthologous putative terpenoid genes Glyma.07G073800.2, Glyma.03G014300.1 and Glyma.07G074600.2 from cultivated soybean (Figure S2) [6]. Moreover, putative subcellular localization studies based on Arabidopsis protein localization for recognized synthesis sites from the Cell eFP database revealed that the *GmFDPS*, *GmGGPPS*, *SgGPS*, *SgFPPS*, and *SgLINS* genes are presents mainly in the cytosol, mitochondrion, nucleus, and plastid (Figure S3). These in silico results align withearlier studies that exhibited all organelles such as cytosol, mitochondrion, nucleus, and plastid can be considered as main loci for terpenoids synthesis and activity [6,8,9,10]. The putative expression patterns and subcellular localization of *GmFDPS*, *GmGGPPS*, *SgGPS*, *SgFPPS*, and *SgLINS* underscore the possible roles of terpenoids in yielding terpene found at infection sites and during infection to attract rhizobia and establish nodulation [6]. Therefore, cloning the full-length cDNA of *GmFDPS*, *GmGGPPS*, *SgGPS*, *SgFPPS*, and *SgLINS* and examining their roles in soybean root and nodule development through overexpressing in hairy root systemsis crucial to proving this hypothesis (Figure 1A and Figure 3A–H). The results demonstrated that this gene plays a significant role in promoting root and nodule growth parameters compared with the GUS control in transgenic G. max hairy roots (Figure 3A–H). Terpenoids and their derivatives have been shown to operate in the root nodules of *G. max* to enhance legume nodulation.

### 4.2. Terpenoid Genes Overexpression Enhances Terpene Accumulation in Transgenic Soybean Hairy Roots

**Figure 9:**
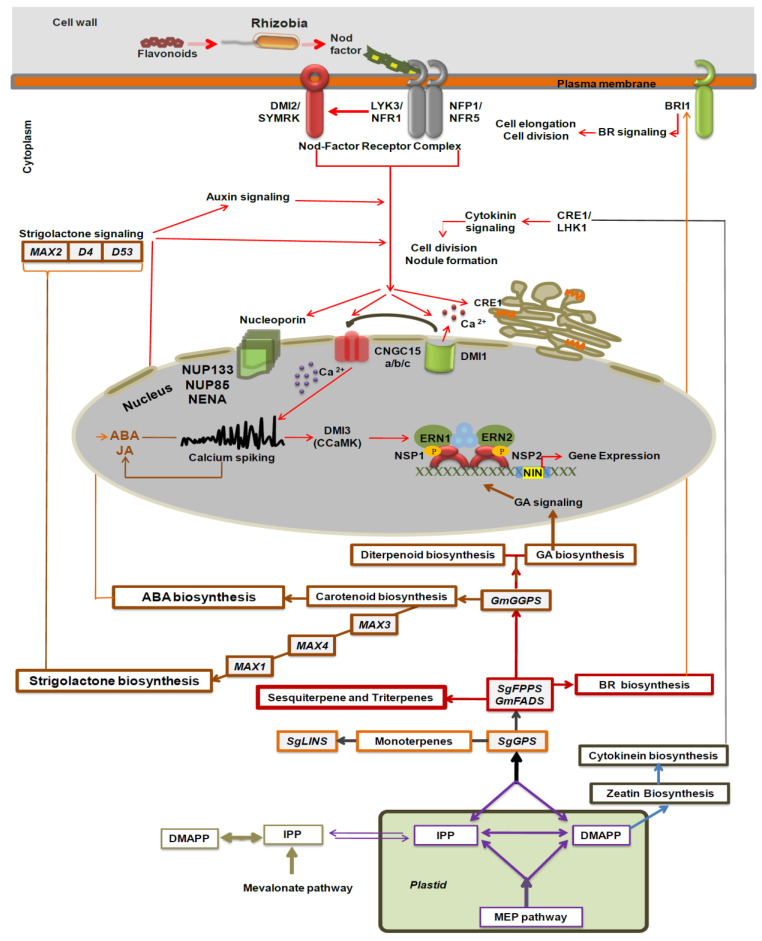
Proposed schematic interactions between terpenoid biosynthesis, terpenoid hormone synthesis, and nodulation pathways in soybean nodulation.Abbreviations:DMAPP: Dimethylallyl diphosphate, IPP: Isopentenyl diphosphate, GPS: geranyl pyrophosphate synthase, FPPS: farnesyl pyrophosphate synthase, GGPPS: geranylgeranyl pyrophosphate synthase, type II, LINA: (3S)-linalool synthase, NSP: Nodulation Signaling Protein, CRE: cytokinin receptor, ERN: ethylene response factor required for nodulation, NIN: nodule inception, CCamK: calcium/calmodulin-dependent protein kinase, BRI1: brassinosteroid insensitive1, CCD7:carotenoid cleavage dioxygenase7, CNGCa/b/c: CNGC: cyclic nucleotide gated channels (a/b/c), NFP: nod factor perception, NFR1: nodfactor receptor, LYK3: LysM receptor kinase, SYMRK: symrk interacting proteins, LHK1: LHK1 cytokinin receptor, DMI: does not make infections, IPD3: interacting proteinof dmi3, NUP85: nucleoporin85, NUP133:nucleoporin133, NENA: transducin/wd40 repeat-like superfamily protein, MAX1, 2, 3 and 4: more axillary branching1, 2, 3 and 4, D4: catotenoid dioxygenase 4, D53 and catotenoid dioxygenase 53.

Remarkably, the overexpression of *GmFDPS*, *GmGGPPS*, *SgGPS*, *SgFPPS*, and *SgLINS* genes enhanced terpene amassing in transgenic soybean hairy roots (Table 1 and Figure 2A,B). For example, mono-, sesqui-, and diterpene compounds were significantly increased in *GmFDPS*, *GmGGPPS*, *SgGPS*, *SgFPPS*, and *SgLINS* transgenic plants compared with the GUS control (Table 1). Moreover, terpene and terpenoid such as Isomenthol (C11952), Pinocarveol (C01767), α-Terpineol (C11393), Isopulegol (C11951), trans-Linalool oxide (C11389), Levomenthol (C00400), phytol (C05427), Farnesan (C09666) play important roles in various pathways (including Monoterpenoid biosynthesis; KEGG: map00902, Biosynthesis of secondary metabolites; KEGG: map01110, Limonene and pinene degradation; KEGG: map00903, Biosynthesis of terpenoids and steroids; KEGG:map01062, Metabolic pathways; KEGG: map01100 and Sesquiterpenoid and triterpenoid biosynthesis; KEGG: map00909) through different biochemical reactions such as (R06420, R03114, R06373, R06374, R06421, R06422, R06417, R06418, R07631, R09702, R09708, R09922, R02177, R02178, R02179, R08530, R08695) (KEGG: https://www.genome.jp/pathway/map00900, https://www.genome.jp/pathway/map00909, https://www.genome.jp/pathway/map00902, https://www.genome.jp/pathway/map00904, accessed on 28 July 2022). Moreover, certain terpenes cause plant development and growth hence considered as primary metabolites rather than secondary metabolites [49] (Table 1 and Figure 9). Furthermore, Carotenoids are mainly one of the terpenoids, and carotenoids such as β-Carotene (C02094) play important roles in various pathways (such as Retinol metabolism; KEGG:map00830, Carotenoid biosynthesis; KEGG: map00906, Biosynthesis of plant secondary metabolites. KEGG: map01060, Biosynthesis of terpenoids and steroids; KEGG: map01062, Biosynthesis of plant hormones; KEGG: map01070, Metabolic pathways; KEGG: map01100, Biosynthesis of secondary metabolites; KEGG: map01110, Biosynthesis of cofactors; KEGG: map01240 and Vitamin digestion and absorption; KEGG:map04977) through various biochemical reactions such as (KEGG: R00032, R03823, R03824, R05345, R07558, R07560, R07857, R08988, R09747, R10282, R10559 and R12179) (KEGG: https://www.genome.jp/pathway/map00830+C02094, https://www.genome.jp/pathway/map00906+C02094, https://www.genome.jp/pathway/map01060+C02094, https://www.genome.jp/pathway/map01062+C02094, https://www.genome.jp/pathway/map01070+C02094, https://www.genome.jp/pathway/map01240+C02094, https://www.genome.jp/pathway/map04977+C02094, accessed on 28 July 2022). Apocarotenoids also include many phytohormones with important functions in plant–environment interactions such as abscisic acid (ABA) and strigolactones (SL) [49] (Table 1 and Figure 9). Particularly, isoprenoids compounds such as, gibberellic acids (GAs), brassinosteroids (BRs), cytokinins (CKs), abscisic acid (ABA) and strigolactones (SLs) (https://www.genome.jp/pathway/map01070+C04691, accessed on 25 March 2021) were reported to affect plant growth, nodule formation, and interaction with other microbial communities [6,21,44,50,51,52] (Figure 9). For instance, these terpenoid hormones are released by roots, such as CKs, SLs, Gas, and BRs, then secreted and transported from the plant root to hairy root cells for root rhizobia interaction, nodule organogenesis and development [21,50,51,52]. So, some of these terpenoid hormones are accumulated upon Nod factor treatment or rhizobia infection, which means they have a central role in legume nodulation and nodule organogenesis [2,27,53,54,55] (Figure 9). The upregulation of the Nod genes is the key player that produces infection thread and root nodules [56,57]. Likewise, terpenoid compounds have been shown to affect nodule formation and root hair patterning in soybean. For example, terpenoid genes such as *SoCINS*, *SoNEOD*, *SoSABS*, *SoLINS*, *SoGPS* and *SoTPS6* from *S. officinalis* affect nodule and root development in soybean transgenic roots, most likely by modulating terpene accumulation [6]. Therefore, that overexpression of *GmFDPS*, *GmGGPPS*, *SgGPS*, *SgFPPS*, and *SgLINS* in transgenic soybean hairy roots has comparable effects on terpene accumulation and nodule formation.

### 4.3. Effect of Terpenoid Genes Overexpressing in Hairy Roots Growth and Soybean Nodulation

The influence of the *GmFDPS*, *GmGGPPS*, *SgGPS*, *SgFPPS*, and *SgLINS* transgene on the expression levels of nodule signaling and SLs biosynthesis genes in transgenic soybean hairy roots and nodules after rhizobial inoculation is critical for understanding their involvement in hairy root development and nodulation. The results revealed that several genes in nodulation signaling and SL synthesis were significantly activated by *GmFDPS, GmGGPPS*, *SgGPS*, *SgFPPS*, and *SgLINS* in hairy roots and root nodules (Figure 5, Figure 6, Figure 7 and Figure 8). The selection of these candidate genes is based on their well-known roles in rhizosphere plant–microbe interactions and nodule development. For example, NFR1 and NFR5 are major nodulation signaling genes in legumes that interact with Nod factors [58,59,60,61,62]. Many studies showed that the interaction of Nod factor receptors (NFRs) with Nod factors is fundamental for early nodule gene expression and nodule organogenesis [6,63,64,65,66,67,68]. Furthermore, Nodule inception transcription factors, *GmNINa* and *GmNINb*, regulate nodule organogenesis and infection thread development [55,69,70]. Moreover, the transcriptional regulators, Nodulation Signaling Pathway, NSP1, and NSP2, upregulate NIN, Early Nodulincoding genes ENOD11 and ENOD40, and Ethylene Response Factor Required for Nodulation1 coding gene (ERN1) throughout Rhizobial infection [63,71,72,73] (Figure 9). Moreover, overexpression of strigolactone biosynthesis genes such as *GmMAX1a, GmMAX3b, GmMAX4a*, and *GmMAX2a* are closely correlated with the augmented nodule number and nodule development, whereas knocking down these genes diminishes nodulation [27,54,55,74].

Ahmad et al. [2] reported that the overexpression of *GmMAX2* in the *G. max* hairy roots system enhances the expression of early nodulation genes such as *DMI2α*, *DMI3α*, *NSP2β*, *NSP1α*, *NFR5α*, and *NFR1α*, but compromised in *GmMAX2* knockdown compared with the control. In addition, hormones such as strigolactones and brassinosteroids are likely to autoregulate nodulationand maintain meristematic activity during nodule development [24,25,27,55,74,75]. In context to that, Ali et al. [6] found that the overexpression of *SoCINS*, *SoNEOD*, *SoSABS*, *SoLINS*, *SoGPS* and *SoTPS6* from *S. officinalis* in the *G. max* hairy roots system enhances the expression of the most nodulation signaling and SL synthesis genes compared with the control. Generally speaking, our findings support that the *GmFDPS*, *GmGGPPS*, *SgGPS*, *SgFPPS*, and *SgLINS* from *G. max* and *S. guaranitica* enhances the transcription of nodulation signaling and SL biosynthesis genes. On the other hand, root growth and nodulation showed different phenotypic plasticity when grow under the same conditions, and the reason behind this phenotypic plasticity may be due to the control of nodulation signaling and SL synthesis genes whose expression is modulated by the overexpression of *GmFDPS*, *GmGGPPS*, *SgGPS*, *SgFPPS*, and *SgLINS* genes [76]. Therefore, these data highlighting *GmFDPS*, *GmGGPPS*, *SgGPS*, *SgFPPS*, and *SgLINS* roles in legume root growth and nodulation are valuable if we harness its benefits to increase legume nodulation, growth, and productivity. In context, the terpenoid genes that we have identified can be used in a gene-editing experiment to augment the value of nodule numbers, fresh weight of nodules, root, and root length [77,78,79,80]. Finally, terpenoid genes donated from wild plant species or landraces plants can be introduced into other cultivated elite lines, for the development ofroot development, and nodulation in soybean and other leguminous plants [77,78,79,80].

## 5. Conclusions

In summary, this study focuses on cloning *GmFDPS*, *GmGGPPS*, *SgGPS*, *SgFPPS*, and *SgLINS* from *G. max* and *S. guaranitica*, over-expressing them in hairy root systems of the cultivated soybean (*G. max*), and assessing the root growth characters and nodulation as has been affected by the transgenic. Transgenic cultivated soybean overexpressing *GmFDPS*, *GmGGPPS*, *SgGPS*, *SgFPPS*, and *SgLINS* presented meaningful changes in root development, nodulation, and the expression levels of nodulation signaling and SL biosynthesis genes. In silico tools and the putative expression analysis were employed to predict *GmFDPS*, *GmGGPPS*, *SgGPS*, *SgFPPS*,and *SgLINS* functions in root and nodule development. Our data affirm that the *GmFDPS*, *GmGGPPS*, *SgGPS*, *SgFPPS*, and *SgLINS* promote root development and nodulation signaling by activating nodulation signaling and SL synthetic genes. As a result, this study provides a clearer vision of the function of the *GmFDPS*, *GmGGPPS*, *SgGPS*, *SgFPPS*, and *SgLINS* in root development and nodulation, in conjunction with nodulation signaling and SL biosynthetic genes that are critical for legume nodulation production.

## Figures and Tables

**Table 1:** The major chemical composition and terpenes from transgenic Soybeanhairyroots.

N	Compound Name	R.T.	Terpene Type	GUS	*GmFDPS*	*GmGGPPS*	*SgGPS*	*SgFPPS*	*SgLINS*
1	(8)Annulene	5.238		0.3	0.94	1.07	3.83	1.59	4.07
2	(−)-Isopulegol	7.376	Mono-					0.02	
3	Artificial Almond Oil	8.382	Organic					0.03	
4	Dihomo-γ-linolenic acid	9.448					0.25	0.03	
5	cis-Verbenol	10.786	Mono-		0.09		0.37	0.13	
6	Dihydrocarveol	14.176	Mono-				0.33	0.12	
7	Limonene dioxide	16.262	Mono-				0.52	0.1	
8	Isomenthol	16.686	Mono-	0.06	0.54	0.37	1.79	0.75	1.44
9	L-trans-Pinocarveol	17.335	Mono-				0.26		
10	Pinocarveol	17.503	Mono-						
11	3-Acetylpyridine	18.997					0.24	0.04	
12	α-Terpineol	19.305	Mono-						
13	Naphthalene	20.446					0.25	0.1	
14	Farnesan	24.283	Sesqui						
15	Indole	24.961					4.57	0.59	
16	Dodecamethylcyclohexasiloxane	25.403			3.09	0.15	1.12		
17	Farnesan	27.982	Sesqui		0.08			0.03	
18	n-Cetane	28.864				0.07		0.15	
19	Caryophyllene oxide	31.027	Sesqui		11.79		3.72	1.3	
20	4-Caranol	31.391	Mono-			0.47	3.59		
21	trans-β-Terpineol	32.127	Mono			0.17		0.29	
22	Isopulegol	35.93	Mono		9.91	3.59	1.29	1.65	
23	β-Eudesmol	36.513	sesqui			0.12			
24	dihydrophytol	38.002	Diter						
25	β-Carotene; β-Carotene, all-trans	39.66							1.35
26	delta.-Cadinol	42.248	Sesqui			0.13	0.76	0.22	
27	Stearic acid; n-Octadecanoic acid;	42.285							
28	Linoleyl alcohol	42.974						0.17	
29	Erucic acid	44.418							7.83
30	Palmitic acid	44.858			3.95	8.01	11.85		60.56
31	trans-Phytol	45.597	Diter			0.49		0.74	
32	Ethyl palmitate	45.617							
33	cis,cis-Linoleic acid	46.84							
34	δ13-cis-Docosenoic acid	46.865			0.55		10.44		
35	Arachic acid	47.235							
36	Phytol	47.305	Diter		9.62	11.6	1.11	12.26	
37	Adamantane, 1,3-dimethyl	48.167					0.65		
38	9-Octadecyne	48.778		0.95					
39	Oleic Acid	49.096					16.88		
40	Stearic acid	49.545			0.66		1.47		2.9
41	trans-Linalool oxide	49.893	Mono-				0.86	0.04	
42	γ-Sitosterol	50.424			11.24	14.66		15.55	
43	dl-Isopulegol	52.064	Mono-					0.27	
44	Mandelic acid	52.514					0.72		
45	Isopropyl linoleate	53.073						0.17	
46	Octadeamethyl-cyclononasiloxane	54.167			8.79		1.19	12.73	
47	Oleyl amide	54.838			0.09	0.09	0.36	0.09	0.49
48	n-Heneicosane	56.512				0.17	1.02	0.16	
49	Methoprene	59.701			6.56	8.04	1.12	10.79	
50	Pulegol	61.087	Mono-			0.09	0.7	0.1	
51	Phthalic acid dioctyl ester	62.27				0.22	0.97	0.46	
52	β,β-Carotene	63.348						0.04	
53	Octadeamethyl-cyclononasiloxane	67.034			6.31	7.47	1.06	10.68	
54	Farnesane	72.172	Sesqui					0.09	
55	Levomenthol	78.19	Mono-			0.07			
Total percentage (%) of monoterpenes				0.6	10.54	4.29	6.12	3.47	1.44
Total percentage (%) of sesquiterpenes					11.87	0.25	4.83	1.72	
Total percentage (%) of diterpenes					9.62	12.09	1.11	13.0	

## Data Availability

All data generated or analyzed during this study are included in this published article and its supplementary information files. The datasets used and/or analyzed during the current study are available from the corresponding author on reasonable request.

## Supplementary Materials

The following supporting information can be downloaded at: https://www.mdpi.com/article/10.3390/cells11172622/s1, Figure S1: Phylogenetic tree of terpenoid genes from *S. guaranitica* with selected terpenoid genes from *G. max* plants. The MEGA6 program was used for the alignment of terpenoid genes through neighbor joining method with bootstrap values based on 1000 replicates; Figure S2: Heat maps representation the putative transcript levels of terpenoid genes from G. max at nine tissues (pod, leaves, root_hairs, root, nodules, seed, sam, stem and flower) from phytozome database (phytozome.jgi.doe.gov/). Green/red color-coded heat maps represent relative transcript levels of different terpenoid and terpene synthases genes in *G. max*, that were determined by alignment of terpenoid genes protein sequences from *S. guaranitica* with *Glycine max* genomic sequences database. MeV: Multi Experiment Viewer software was used to depict transcript levels; Figure S3: Putative subcellular localisations of terpenoid genes based on Arabidopsis protein localization at different cell organs. Cell sub-cellular localisations profile images were built using Cell ElectronicFluorescent Pictograph Browsers (Cell eFP browsers. The blue arrow points the expression scale (the more intense red color, the more gene expression), http://bar.utoronto.ca/cell_efp/cgi-bin/cell_efp.cgi; Table S1: List of *Glycine max* and *S. guaranitica* genes and primer pairs used for full-length terpene synthases cDNAs clones; Table S2: List of *Glycine max* and *S. guaranitica* genes and primer pairs used for qRT-PCR; Table S3: List of Glycine max genes involved in nodules biosynthesis and signaling pathway and primer pairs used for qRT-PCR.
