# Supplementary material for: Overexpression of Terpenoid Biosynthesis Genes Modifies Root Growth and Nodulation in Soybean (Glycine max)
Source: Cells. 2022 Aug 23;11(17):2622. doi: 10.3390/cells11172622 (PMC9454526; doi:10.3390/cells11172622)
Supplement: Supplementary file 1 [file cells-11-02622-s001.zip › cells-1817995-supplementary/cells-1817995-supplementary figures.pdf]

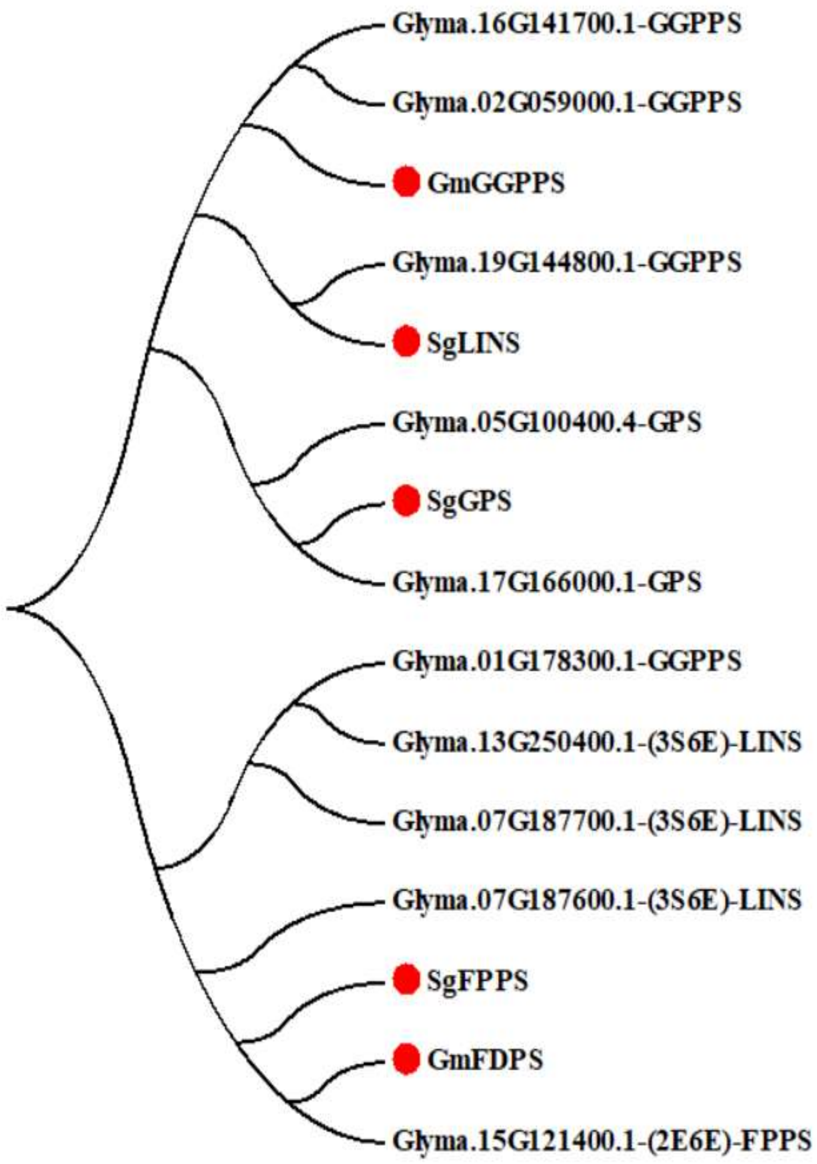

Figure S1: Phylogenetic tree of terpenoid genes from *S. guaranitica* with selected terpenoid genes from *G. max* plants. The MEGA6 program was used for the alignment of terpenoid genes through neighbor joining method with bootstrap values based on 1000 replicates.

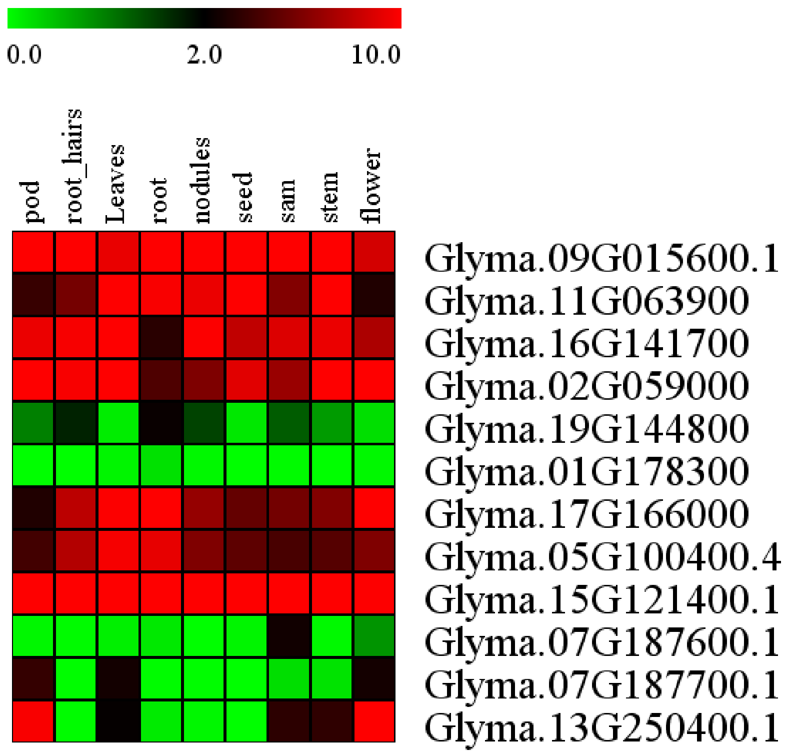

Figure S2. Heat maps representation the putative transcript levels of terpenoid genes from *G. max* at nine tissues (pod, leaves, root_hairs, root, nodules, seed, sam, stem and flower) from phytozome database (phytozome.jgi.doe.gov/). Green/red color-coded heat maps represent relative transcript levels of different terpenoid and terpene synthases genes in *G. max*, that were determined by alignment of terpenoid genes protein sequences from *S. guaranitica* with *Glycine max* genomic sequences database. MeV: Multi Experiment Viewer software was used to depict transcript levels.

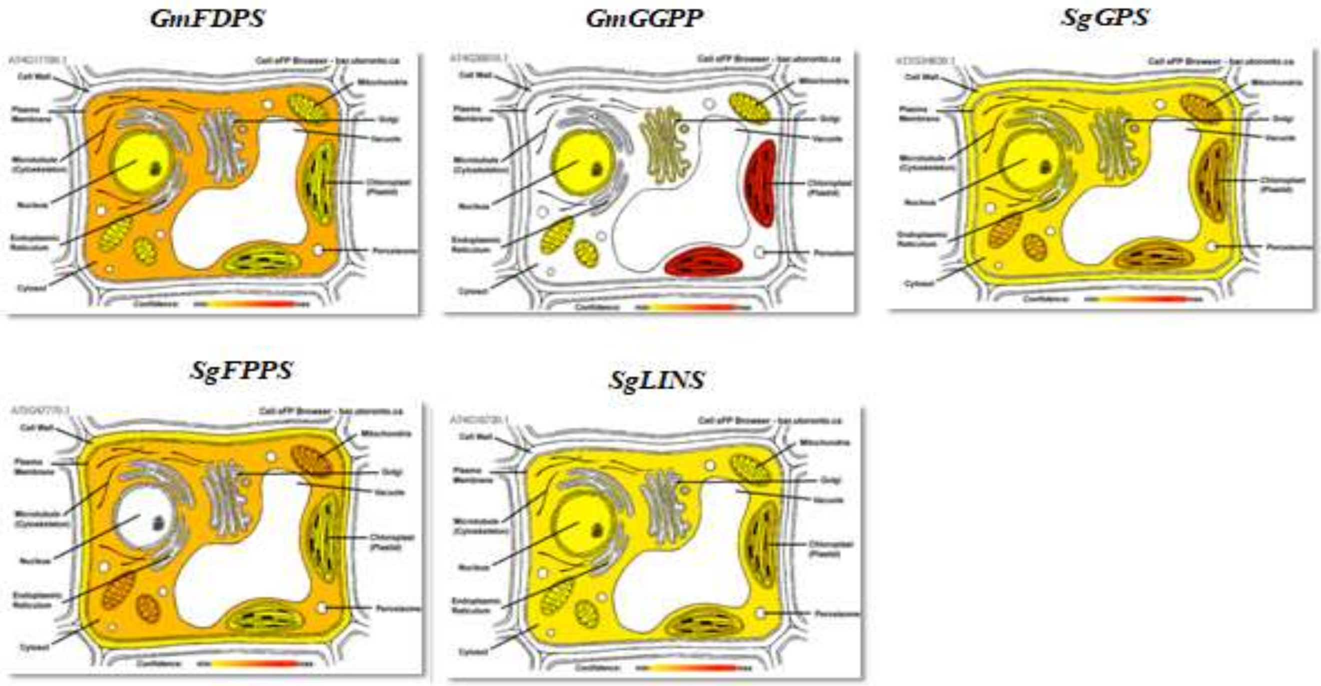

Figure S3. Putative subcellular localisations of terpenoid genes based on Arabidopsis protein localization at different cell organs. Cell sub-cellular localisations profile images were built using Cell Electronic Fluorescent Pictograph Browsers (Cell eFP browsers. The blue arrow points the expression scale (the more intense red color, the more gene expression), http://bar.utoronto.ca/cell_efp/cgi-bin/cell_efp.cgi
