# Supplementary material for: Overexpression of Terpenoid Biosynthesis Genes Modifies Root Growth and Nodulation in Soybean (Glycine max)
Source: Cells. 2022 Aug 23;11(17):2622. doi: 10.3390/cells11172622 (PMC9454526; doi:10.3390/cells11172622)
Supplement: Supplementary file 1 [file cells-11-02622-s001.zip › cells-1817995-supplementary/cells-1817995-supplementary tables.pdf]

Table S1. List of *Glycine max* and *S. guaranitica* genes and primer pairs used for full-length terpene synthases cDNAs clones.

Gene name	Short primer sequence	Long primer sequence
GmFDPS	5'- ATGGCAGATCTCAAGTCTACATTC-3'	5'- GGGGACAAGTTTGTACAAAAAAGCAGGCTTATGGCAGATCTCAAGTCT-3'
	5'- CCTTAAGTTGACTCTACTTCTGCCT-3'	5'- GGGGACCACTTTGTACAAGAAAGCTGGGTTTGACTCTACTTCTGCCT-3'
GmGGPP	5'- GCACCAAACATGAGTTCTCTGAATC-3'	5'- GGGGACAAGTTTGTACAAAAAAGCAGGCTTGCACCAAACATGAGTTCT-3'
	5'- GTGAGAAAACAGGGTATCCAATGTT-3'	5'- GGGGACCACTTTGTACAAGAAAGCTGGGTCAGGGTATCCAATGTT-3'
SgLINS	5'- ATGCTGATGAAAACAGAGCAAGATTC-3'	5'- GGGGACAAGTTTGTACAAAAAAGCAGGCTTCATGCTGATGAAAACAGAG-3'
	5'- TTAAATGAATTTCAAGGCATTTCGAG-3'	5'- GGGGACCACTTTGTACAAGAAAGCTGGGTTTAAATGAATTTCAAGGC-3'
SgGPS	5'- ATGATGTCGGTTAGAGGGCTCG-3'	5'- GGGGACAAGTTTGTACAAAAAAGCAGGCTTCATGATGTCGGTTAGAGGG-3'
	5'- CTACTTTGTTCTGGTGATGACTATATGCG-3'	5'- GGGGACCACTTTGTACAAGAAAGCTGGGTCTACTTTGTTCTGGTGAT-3'
SgFPPS	5'- ATGGCGAATCTCAACGGATC-3'	5'- GGGGACAAGTTTGTACAAAAAAGCAGGCTTCATGGCGAATCTCAACG-3'
	5'- TTATTTCTGTCTCTTGTATATCTTTCCC-3'	5'- GGGGACCACTTTGTACAAGAAAGCTGGGTTTATTTCTGTCTCTTG-3'

Table S2. List of *Glycine max* and *S. guaranitica* genes and primer pairs used for qRT-PCR.

Gene	Primer name	Primer sequence	PCR product (bp)
GmFDPS	GmFDPS-F	5'- TGAATGGCTTCAGGCATATT-3'	151
	GmFDPS-R	5'- GATCCGTGGAATATGGTTTCT-3'	
GmGGPP	GmGGPP-F	5'- AACAACGCTTCCCAAACT-3'	146
	GmGGPP-R	5'- CGATCATGTAGCCCTTGAAA-3'	
SgGPS	SgGPS-F	5'- GCTTGAGGTCTATGGTAGTTG -3'	155
	SgGPS -R	5'- CTCGGATGTTTGCCTCTG -3'	
SgFPPS	SgFPPS-F	5'- CACACAAGACGTGGTCAG -3'	159
	SgFPPS-R	5'- CCAACAGATCGACATAGTAAGG -3'	
SgLINS	SgLINS -1-F	5'- GGTGAAGGCTGAGGAATATC -3'	156
	SgLINS -1-R	5'- AGTTTGGCGACGGAATG -3'	
GmACTIN	GmACTIN-F	5'- CTTCCCTCAGCACCTTCCAA-3'	158
	GmACTIN-R	5'- GGTCCAGCTTTCACACTCCAT-3'	

Table S3. List of *Glycine max* genes involved in nodules biosynthesis and signaling pathway and primer pairs used for qRT-PCR.

Gene name	Primer name	Primer sequence	PCR product (bp)
GmMAX1a	*GmMAX1a-F*	5'- CCTCTCTAGTCCCCATTCAGTTT-3'	150
	GmMAX1a-R	5'- TGTGTTGGTTGATGAAATCTGATAC-3'	
GmMAX1b	*GmMAX1b-F*	5'- TACCGCAAATATTCTTCAGACC-3'	155
	GmMAX1b-R	5'- ACCAGATTCTATCCCTTTCACC-3'	
GmMAX2	*GmMAX2-F*	5'- TGTTTGCTCGCCAGGACTC-3'	160
	GmMAX2-R	5'- CACCATCACCCGGTTCATC-3'	
GmMAX3	*GmMAX3-F*	5'- CAGGCTACTCACCGTGTCTT-3'	152
	GmMAX3-R	5'- GGTATCCGTTACAGCCCAAT-3'	
GmMAX4a	*GmMAX4a-F*	5'- CTTTCACCACCTTGCCTTCA-3'	148
	GmMAX4a-R	5'- TGGTTTCTTTCCGTTCCTCC-3'	
GmMAX4b	*GmMAX4b-F*	5'- GAAATAAAGGCATCTACAAAGGGAA-3'	150
	GmMAX4b-R	5'- ATGGTGGTGGTGGTGGCAGT-3'	
GmNINa	*GmNINa-F*	5'- TAACATGCGATGCTGATCTTG-3'	150
	GmNINa-R	5'- TGATTTAGAGGCGAAGCTTGA-3'	
GmNINb	*GmNINb-F-*	5'- CATGGAGTCGACGCAAATAA-3'	160
	GmNINb-R	5'- TCAAGTACCCAACAGCAATC-3'	
GmNFR5	*GmNFR5-F*	5'- TTCCCTTTCTTCCTCTCCAC-3'	149
	GmNFR5-R	5'- GCATGAAAAGTTTGTTCTATTGTC-3'	
GmNSP1a	*GmNSP1a-F*	5'- CAACACTTATCTTCTTCTCCAACT-3'	160
	GmNSP1a-R	5'- GGAAGCATTTGCTATGTTGTTAGG-3'	
GmNSP1b	*GmNSP1b-F*	5'- ATCCTCGTCTTCTTCCAAATAC-3'	155
	GmNSP1b-R	5'- GGGAGAAGGAGTAGGAGTAAT-3'	
GmNSP2a	*GmNSP2a-F*	5'- GAACTTACCGCACCTTAGTT-3'	155
	GmNSP2a-R	5'- GATGCAGCGACTCCATAAA-3'	
GmNSP2b	*GmNSP2b-F*	5'- AATCATTGCCAAGCGAAGCT-3'	149
	GmNSP2b-R	5'- AGTCCAAAGCGAGGCAGAGA-3'	
GmNSP2b	*GmDMI2a-F*	5'- GTCCTCAGTGGCCTTGACATT-3'	160
	GmDMI2a-R	5'- ACACCCTTTTGCCTGCTTTG-3'	
GmDMI2b	*GmDMI2b-F*	5'- ATTCACGAGCACACTGTGCCT-3'	158
	GmDMI2b-R	5'- CCAAAATCTGCAACCTTTCC-3'	
GmDMI3α	*GmDMI3α-F*	5'- AGTGTTTGGAGCACCGCAATC-3'	147
	GmDMI3α-R	5'- TCAAACAAGTCAAATATACGTGGTG-3'	

GmDMI3b	*GmDMI3b-F*	5'- TTGTCCATAGGGAGGGTAAT-3'	159
	GmDMI3b-R	5'- ATGAGTAGAAGGGTAGCTAGAG-3'	
GmACTIN	*GmACTIN-F*	5'- CTTCCCTCAGCACCTTCCAA-3'	158
	GmACTIN-R	5'- GGTCCAGCTTTCACACTCCAT-3'	
